# Fine Mapping of a Major Locus for Leaf Sheath Hairiness in Wheat Identifies *TaSAIN1-4D* as a Candidate Gene

**DOI:** 10.3390/genes16091117

**Published:** 2025-09-20

**Authors:** Lijuan Wu, Jundong He, Shian Shen, Yulin Li, Jinbai He, Xinkun Hu

**Affiliations:** 1 Institute of Ecology, China West Normal University, Nanchong 637009, China; wulijuan0921@126.com (L.W.); jundhe@163.com (J.H.); shenshianfm@126.com (S.S.); 2 College of Agronomy, Sichuan Agricultural University, Chengdu 611130, China; 3 College of Life Science, China West Normal University, Nanchong 637009, China; 18781774286@163.com (Y.L.); 13696004121@163.com (J.H.)

**Keywords:** *Triticum aestivum* L., leaf-sheath pubescence, quantitative trait locus (QTL), trichome development, stress tolerance, *TaSAIN1-4D*, KASP marker

## Abstract

**Background/Objectives**: Leaf sheath hairiness (LSH) is an adaptive trait in wheat that improves tolerance to biotic and abiotic stresses. Although trichome development has been extensively studied in model plants, the genetic basis of LSH in Triticeae crops remains poorly defined. **Methods**: In this study, the inheritance and genetic architecture of LSH were investigated. Two F_2_ populations were used, derived from crosses between the glabrous lines ‘Shumai 830’ and ‘Shumai 2262’ and the hairy line ‘Zhongkelanmai 1’. BSA-seq was combined with KASP marker genotyping to map and refine the trait locus. Candidate genes were evaluated through comparative genomics; sequence variation; and subcellular localization prediction. **Results**: Phenotypic evaluation revealed that LSH is a dominant trait, segregating at a 3:1 ratio in F_2_ populations. BSA-seq identified a major locus, *QLsh.cwnu-4D*, on chromosome 4DL. Fine mapping with KASP markers refined this region to a 1.67 Mb interval overlapping a 530 kb trichome-associated linkage disequilibrium block in *Aegilops tauschii*. Within this interval, *TaSAIN1-4D*, a salt-inducible protein unique to Triticeae, was identified as the strongest candidate gene. Extensive sequence variation among alleles (*TaSAIN1-4Da*; *TaSAIN1-4Db*; *TaSAIN1-4Dc*), including large insertions and multiple SNPs, indicated potential functional diversification. Predicted nuclear localization of *TaSAIN1-4D* supports a role in trichome regulation and stress adaptation. The co-dominant KASP marker *K-cwnu-4D-502238348* was tightly linked to LSH and cosegregated perfectly, making it a reliable tool for marker-assisted selection. **Conclusions**: This study clarifies the genetic architecture of leaf sheath hairiness in wheat and identifies *TaSAIN1-4D* as a likely regulator. These findings provide a practical marker-assisted selection tool that can accelerate the development of improved wheat varieties with desirable leaf surface traits.

## 1. Introduction

Leaf pubescence, characterized by the presence of trichomes or leaf hairs (LHs), is a common adaptive trait in angiosperms, including major cereal crops such as wheat, barley, rye, and rice [1,2]. These epidermal outgrowths range from simple unicellular projections to complex multicellular structures, with various morphologies, including straight, hooked, branched, or spiral forms. Glandular trichomes secrete secondary metabolites (e.g., terpenoids, alkaloids, phenolics, and phenylpropanoids) that deter herbivores and inhibit pathogen colonization [3]. In contrast, non-glandular trichomes enhance abiotic stress tolerance by reducing transpirational water loss, modulating leaf surface temperature, and improving moisture retention during drought conditions [4,5].

In Triticeae, natural variation in trichome traits is controlled by multiple quantitative trait loci (QTLs). In bread wheat (*Triticum aestivum* L.), the dominant gene *Hl1* on chromosome 4BL governs medium-length trichome formation [4,6], while additional QTLs on 4BS, 7BS, 7D, and 4DL influence trichome density and distribution [7,8]. In barley (*Hordeum vulgare* L.), leaf-blade and sheath pubescence are mapped to chromosomes 3HL and 4HL, respectively [9,10]. The barley Hairy leaf sheath (*Hsh*) gene on 4HL is collinear with the rye Hairy peduncle 1 (*Hp1*, 5RL) and wheat *Hl1* (4BL) [7,11,12]. In the D-genome progenitor *Ae. tauschii*, known for its dense auricle and leaf hairs, candidate loci for trichome traits include *hfl* on 3D and *Hsh* on 4DL [8,13,14]. A genome-wide association study of 242 accessions also identified a 530 kb linkage disequilibrium (LD) block on 4DL associated with marginal trichome number [15].

Breeding efforts have successfully introgressed novel trichome alleles from wild relatives into cultivated wheat. A prominent example is the introgression of an *Hl1* variant (*Hl1^th^*) from *Thinopyrum ponticum* (2n = 10x = 70, StStStStE^e^E^e^E^b^E^b^E^x^E^x^) into the wheat cultivar ‘Saratovskaya 29’ via chromosome 4th substitution. This has enabled marker-assisted dissection of gene interactions and the development of stress-resilient, high-yielding wheat lines [16]. Despite these advances, the primary genes controlling leaf pubescence in Triticeae remain unmapped and uncloned, limiting functional studies and practical breeding applications.

The present study employs bulked segregant analysis sequencing (BSA-seq) to map QTL for leaf-sheath hairiness (LSH) in two F_2_ populations derived from crosses between glabrous lines ‘Shumai 830’ and ‘Shumai 2262’ and the hairy line ‘Zhongkelanmai 1’, which carries the genetic lineage of ‘Chuanmai 42’ (CM42) and *Th. ponticum*, and may harbor *QLsh.saas-4D* or *Hl1^th^* [8,16]. Resultly, the *Lsh* locus were delimited to a 2.11 Mb interval overlapping the 530 kb *Ae. tauschii* LD block, refine it to 1.67 Mb with Kompetitive Allele-Specific PCR (KASP) markers, and conduct molecular cloning, sequence analysis, and subcellular localization prediction of candidate genes. This work lays the groundwork for cloning the key LSH gene and its potential deployment in wheat improvement.

## 2. Materials and Methods

### 2.1. Plant Materials

**Figure 1:**
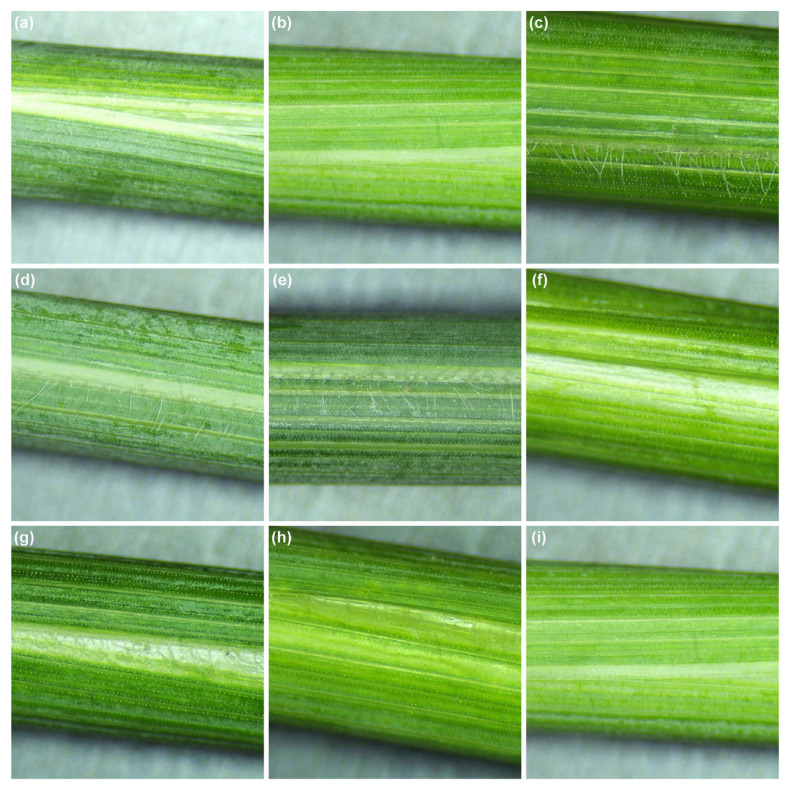
Phenotypic of the leaf sheath. (**a**) SM830; (**b**) SM2262; (**c**) ZKLM1; (**d**) F_1_ hybrid of the SM830/ZKLM1 cross; (**e**, **f**) LSH phenotypes in the SM830/ZKLM1 F_2_ population; (**g**) F_1_ hybrid of the SM2262/ZKLM1 cross; (**h**, **i**) LSH phenotypes in the SM2262/ZKLM1 F_2_ population.

Three hexaploid wheat cultivars were utilized to generate two F_2_ segregating populations for BSA-seq analysis. The cultivars Shumai 830 (SM830; pedigree: SHW-L1/Chuannong 16//Pm99915-1/3/03-DH1959) and Shumai 2262 (SM2262; pedigree unknown) both exhibit glabrous leaf sheaths and blades, though SM830 retains a hairy auricle (Figure 1, Figures S1 and S2). These served as the female parents and were developed at the Triticeae Research Institute, Sichuan Agricultural University. The male parent, Zhongkelanmai 1 (ZKLM1; pedigree: Chuanmai 42/Lannuoxiaomai 12//R64002), displays a hairy leaf sheath, glabrous leaf blade, auricle, and blue grains (Figure 1, Figures S1 and S2). The blue grain trait in ‘Lannuoxiaomai 12’ originates from wheat–*Th. ponticum* 4EL chromosomal translocation lines. ZKLM1 was bred at the Chengdu Institute of Biology, Chinese Academy of Sciences. Crosses between SM830 × ZKLM1 and SM2262 × ZKLM1 produced two F_2_ populations (hereafter referred to as ‘SM830/ZKLM1’ and ‘SM2262/ZKLM1’) for subsequent bulked-segregant analysis.

### 2.2. Field Trial and Growth Conditions

The two F_2_ populations, along with their parental lines, were evaluated during the 2024–2025 growing season at the experimental farm of China West Normal University in Nanchong, Sichuan Province. Each genotype was sown in a 1.5 m single row, with 15 seedlings per row and 0.3 m between adjacent rows. Standard agronomic practices for wheat, including land preparation, irrigation, and fertilization, were followed throughout the season.

### 2.3. Bulk Construction and Targeted Genotyping by Sequencing

LSH is a qualitative trait, which enabled phenotyping of the F_2_ segregating populations by visual inspection. From the SM830/ZKLM1 F_2_ population, 52 LSH+ and 55 LSH− (approximately 1:1) individuals were selected to construct one mixed pool, while from the SM2262/ZKLM1 F_2_ population, 56 LSH+ and 28 LSH− (2:1) individuals formed a second mixed pool. Flag leaf samples were collected using a 6 mm punch, with five leaf discs taken from each plant within both pools and the parental lines. Genomic DNA was extracted using the Plant Genomic DNA Kit (DP305, Tiangen Biotech, Beijing, China). DNA quality was assessed by agarose gel electrophoresis (1%) and quantified using the Qubit Flex fluorometer (ThermoFisher Scientific, Waltham, MA, USA).

Genomic DNA from the mixed pools and parental lines was sent for 120K-4HWA SNP probe-targeted genotyping by sequencing (Tcuni, Chengdu, China). DNA libraries were constructed, probes captured, and quality controlled following Tcuni’s standard procedures. Paired-end sequencing was conducted on the DNBSEQ-T7 platform (MGI Tech, Shenzhen, China), with sequencing depths of 60× for the mixed pools and 30× for the parental lines. The paired-end sequencing comprised two rounds: the first round captured the template strand, and the second round obtained the complementary strand.

### 2.4. Data Processing and Variant Calling

The data analysis pipeline included raw data quality control, data alignment, variant calling, and statistical analysis. Raw sequencing reads were processed using the fastp software (version 0.23.4, parameters: -n 15 -q 15 -u 40) to ensure data quality [17]. The processing steps included: (1) removal of adapter sequences; (2) exclusion of paired reads containing more than 15 ‘N’ bases; and (3) exclusion of paired reads with more than 40% bases with quality scores (Q) ≤ 15. Clean reads were aligned to the wheat cv. Chinese Spring reference genome (RefSeq v2.1) using the MEM algorithm in BWA (v0.7.17) [18,19]. SNPs and InDels were called using GATK [20], and variants with low confidence were filtered out. High-confidence variants were annotated, and their potential effects were predicted using SnpEff [21].

### 2.5. BSA-Seq Analysis

Raw reads were first subjected to quality control using fastp v0.23.4. Low-quality bases (Phred score < 30) and adapter sequences were removed, and reads shorter than 50 bp after trimming were discarded. Approximately 0.61% of reads were filtered out on average across the samples. QTL identification was conducted using three complementary methods, including the Euclidean distance (ED) algorithm, the *G′* statistic, and the classical Δ(SNP-index) approach [22,23,24]. For the ED analysis, SNPs exceeding the 99% confidence interval were retained, and their ED values were smoothed to highlight significant peaks. The QTLseqr R package v0.7.5.2 was employed to compute both the SNP-index and *G′* statistic with a 2 Mb sliding window and 10 kb steps. Statistical significance was assessed at *p* < 0.05 after Bonferroni correction [25]. Candidate QTL regions were defined as genomic intervals consistently detected by all three methods. Genes within these overlapping intervals were annotated using the intervalTools utility on the WheatOmics 1.0 platform (http://202.194.139.32/tools/intervalTools.html, accessed on 16 June 2025).

### 2.6. KASP Marker Development and Fine Mapping

To validate and fine-map the identified QTL, 92 F_2:3_ family lines exhibiting extreme LSH phenotypes were selected from the SM830 × ZKLM1 F_2_ population for KASP analysis. Genomic DNA from these F_2:3_ lines and their parents was isolated as described above. KASP markers were designed based on differential SNP probes within the QTL regions, with each marker corresponding to a specific SNP locus. Primer sequences for KASP markers were designed using the PrimerServer tool on the WheatOmics 1.0 platform (http://202.194.139.32/snprimer/, accessed on 20 June 2025). Two allele-specific forward primers and a common reverse primer were synthesized by Sangon Biotech (Shanghai, China) (Table S1). Marker specificity was first confirmed between the parental lines and then genotyped across the 92 F_2:3_ family lines. Genotyping was performed following the protocol of Liu et al. (2024) on a BIO-RAD CFX Connect™ Real-Time PCR System (Bio-Rad, Hercules, CA, USA) [26]. Across all assays, the average call rate exceeded 98%, and the reproducibility between replicates was greater than 99%, indicating high reliability of the developed markers for fine mapping.

### 2.7. Molecular Cloning and Sequence Analysis of Candidate Genes

Total RNA was isolated from flag leaves of SM830 and ZKLM1 using the TaKaRa MiniBEST Plant RNA Extraction Kit (TaKaRa, Dalian, China). First-strand cDNA was synthesized with the PrimeScript RT reagent Kit (TaKaRa) according to the manufacturer’s instructions. PCR amplifications were performed on cDNA and genomic DNA templates to obtain both coding sequences (CDS) and genomic sequences of each candidate gene, following the conditions and cloning workflow described by Hu et al. (2018) [27]. Amplified fragments were purified, cloned into the pJET1.2/blunt vector (Thermo Fisher, Waltham, MA, USA), and three independent positive clones per target were sequenced by Sangon Biotech (Shanghai, China). Sequence assembly and alignment were conducted in DNAMAN 8.0. Signal peptides were predicted using SignalP v6.0 (https://services.healthtech.dtu.dk/services/SignalP-6.0/, accessed on 25 July 2025), and subcellular localization was inferred using DeepLoc v2.1 (https://services.healthtech.dtu.dk/services/DeepLoc-2.1/, accessed on 25 July 2025). All primer sequences are provided in Table S1.

## 3. Results

### 3.1. Phenotypic Evaluation

The phenotypic evaluation of the F_1_ hybrids, F_2_ populations, and their parental lines was carried out through visual observation of leaf sheath and auricle hairiness. The parental lines SM830 and SM2262 were both glabrous for the leaf sheath and blade. In contrast, ZKLM1 exhibited a distinct hairy leaf sheath phenotype (Figure 1a–c). Upon crossing these lines, both F_1_ hybrids, SM830/ZKLM1 and SM2262/ZKLM1, displayed leaf sheath hairiness (Figure 1d,g). This confirmed that the hairy leaf sheath trait is dominant over the glabrous phenotype. In the F_2_ populations derived from the SM830/ZKLM1 and SM2262/ZKLM1 crosses, the segregation of the LSH trait followed a 3:1 ratio, with approximately three-quarters of the plants showing the LSH phenotype and one-quarter exhibiting a glabrous leaf sheath (Figure 1e,h for LSH+ plants; Figure 1f,i for glabrous plants). For auricle hairiness, SM830 had hairy auricles, while SM2262 and ZKLM1 exhibited glabrous auricles (Figure S1a–c). The F_1_ hybrids from cross SM830/ZKLM1 exhibited hairy auricles, and approximately 75% of the F_2_ individuals from populations displayed auricle hairiness, irrespective of their leaf sheath phenotype (Figure S1d–f). Notably, all tested lines, including both parents and hybrids, had glabrous leaf blades (Figure S2).

### 3.2. Characterization of Targeted Capture Sequencing Data

To identify QTLs associated with LSH, four bulk-segregant pools (two for each F_2_ population) and the three parental lines were genotyped using a 120K-4HWA SNP-capture sequencing. A total of 297,110,842 raw reads (41.51 Gb) were generated during sequencing. After quality trimming, 295,242,880 clean reads (40.19 Gb) were retained, representing 99.30% to 99.51% of the total reads, with Q30 scores consistently exceeding 96%. The GC content ranged from 51.1% to 52.9%, with an average of 52.3%. Duplicate reads were kept below 4%, ensuring high-quality data (Table 1).

Mapping the clean reads to the wheat ‘Chinese Spring’ reference genome (RefSeq v2.1) resulted in an alignment rate of 99.99%. The mean sequencing coverage depths were ≥60× for the pooled samples and ≥30× for the parental lines. The capture rate of the four bulks ranged from 30.85% to 31.30%, while the capture rates of the parents ZKLM1 and SM830 were close to 30%, at 28.53% and 29.89%, respectively. In contrast, the capture rate of the parent SM2262 was lower, at only 22.84%. Regions covered by at least 10 reads (coverage ≥ 10×) accounted for over 94% in all pools. However, coverage was slightly lower for individual parental lines: 87.59% for ZKLM1, 87.45% for SM830, and 82.16% for SM2262 (Table 2). Nevertheless, because the coverage ≥10× exceeded 80% in all samples, the datasets were considered suitable for downstream Bulked-Segregant Analysis (BSA).

### 3.3. BSA-Seq Analysis and QTL Identification

BSA was performed on the two F_2_ bulk-segregant pools to map QTLs associated with the LSH trait. To reduce background noise and focus on the target signal, the three parental lines were included in the analysis. Three complementary methods were used for association mapping and QTL detection, namely the ED approach, the *G′* statistic, and the Δ(SNP-index) method.

**Figure 2:**
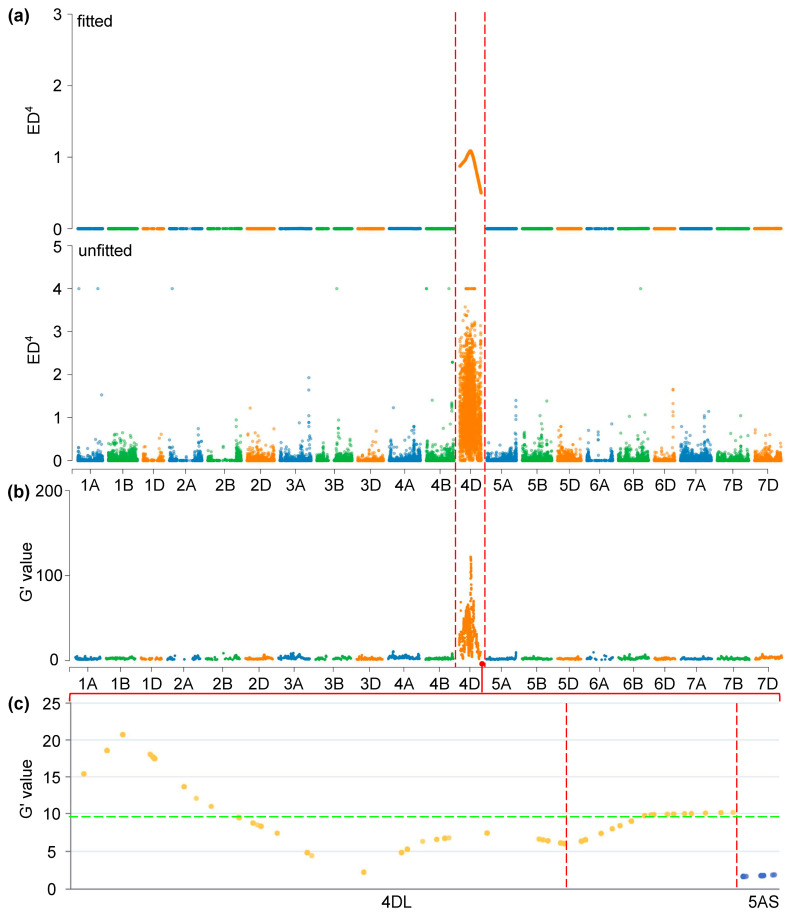
QTL mapping of the LSH trait in SM830/ZKLM1 bulks using Euclidean distance (ED)- and *G′*-based metrics. (**a**) Manhattan plot of unfitted ED^4^ and locally fitted ED^4^ values across all 21 wheat chromosomes, highlighting peaks corresponding to trait-associated loci. (**b**) Genome-wide *G′* profile showing smoothed allele frequency divergence between bulks. (**c**) An enlarged view of the *G′* profile for the distal region of chromosome 4D. *G′* statistics were computed from SNP-wise G values using a sliding window smoothing method. Significance thresholds (*G′* ≥ 9.714; *n* = 107) were assessed using empirical permutation testing and indicated using green dotted line.

For the ED analysis, raw ED values were raised to the fourth power (ED^4^) and smoothed using the SNPNUM method. The significance threshold for the smoothed ED^4^ values was set at 0.2. In both the SM830/ZKLM1 F_2_ bulk-segregant pools, a strong peak in ED^4^ values was detected on chromosome 4D, reaching approximately 1.0 in one pool and around 0.5 in the other, while all other chromosomes remained near zero (Figure 2a and Figure S3a). This unambiguously localized the LSH-associated QTL to chromosome 4D.

**Figure 3:**
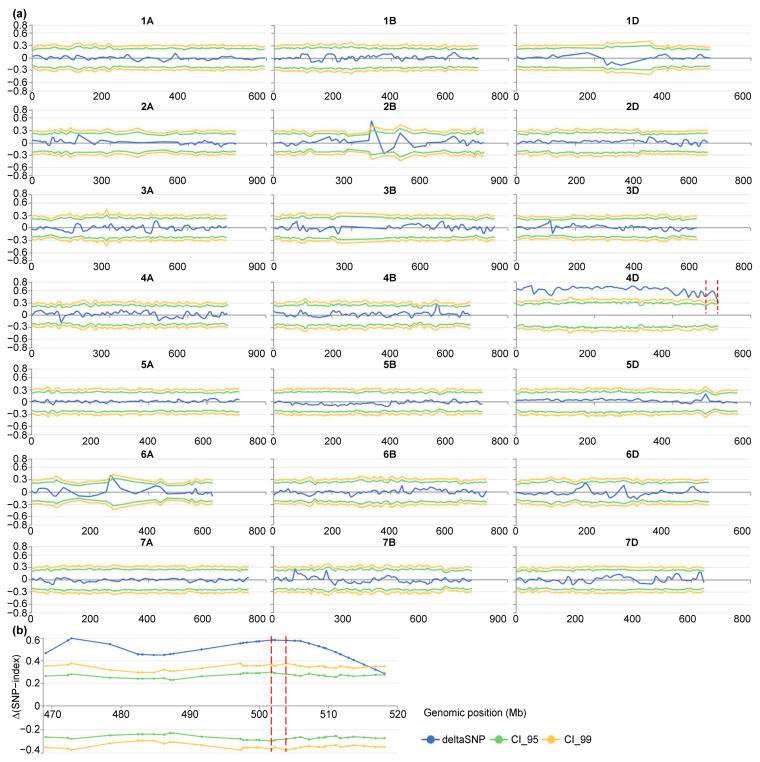
QTL analysis of the LSH trait based on the Δ(SNP-index) algorithm applied to mixed pools of SM830/ZKLM1. (**a**) Line charts depict the trend of Δ(SNP-index) across all 21 chromosomes, and (**b**) an enlarged view of the distal region of chromosome 4D highlights a major QTL peak. The blue, green, and yellow lines represent the Δ(SNP-index), 95% confidence interval (CI), and 99% CI, respectively. CI (*n* = 107) were determined via simulation-based null distribution modeling. Significant Δ(SNP-index) peaks exceeding the 95% or 99% CI indicate potential QTLs. The red dashed line marks the genomic interval most strongly associated with the LSH trait.

The *G*′ statistic corroborated the findings from the ED analysis, with a significant signal on chromosome 4D in both bulks (Figure 2b and Figure S3b). Additionally, the Δ(SNP-index) method further refined the localization, identifying significant intervals on the long arm of chromosome 4D (4DL). Four significant intervals were observed in one bulk, while only two were detected in the other (Figure 3a and Figure S4a).

By intersecting these intervals with previously reported LSH QTLs [8] and recent GWAS hits for leaf-margin trichomes [15], a consensus QTL region was identified at the distal end of chromosome 4DL. This region was designated *QLsh.cwnu-4D* (Figure 2c, Figure 3b, Figures S3c and S4b). The peak of this QTL spanned a 2.11 Mb interval (Chinese Spring_RefSeq v2.1: n.501803422–n.503905439), which is a strong candidate region for the LSH trait (Table 3).

### 3.4. KASP Marker-Based Fine Mapping of LSH Traits

**Figure 4:**
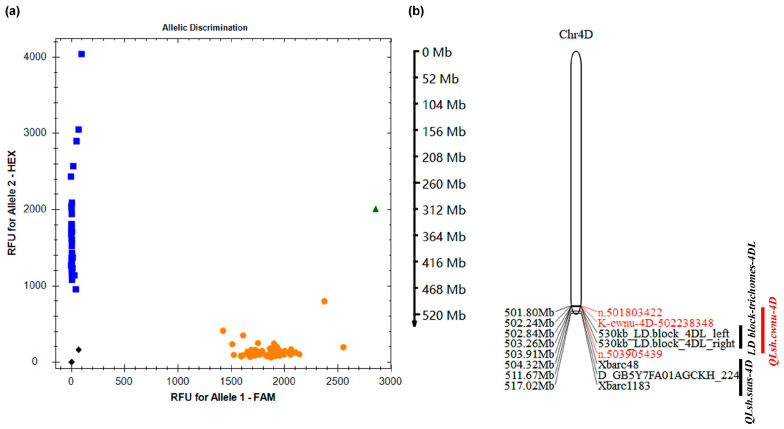
KASP marker analysis and construction of the physical map. (**a**) Genotyping of 92 F_2:3_ family lines with extreme phenotypes using KASP markers. (**b**) Physical map depicting the major QTLs associated with LSH or trichome traits. Circles, squares, triangles, and diamonds represent allele 1, allele 2, heterozygous genotypes, and non-template control (NTC), respectively.

To fine-map the QTL associated with LSH, KASP markers were designed based on the SNPs identified in the *QLsh.cwnu-4D* region. These markers were then used to genotype a panel of 92 F_2:3_ family lines exhibiting extreme phenotypes for LSH from the SM830 × ZKLM1 population. Among the markers, *K-cwnu-4D-502238348* produced a clear, co-dominant genotype that perfectly co-segregated with the LSH trait (Figure 4a). This marker helped narrow down the *QLsh.cwnu-4D* region to a 1.67 Mb segment, bounded by *K-cwnu-4D-502238348* and SNP *n.503905439* (502,238,348–503,905,439 bp). This refined interval overlapped a 530 kb trichome-associated LD block in *Ae. tauschii* and is adjacent to the previously identified QTL for LSH in the wheat cv. Chuanmai 42 (CM42) (Figure 4b).

### 3.5. Gene Prediction Within QLsh.cwnu-4D

Gene models within the *QLsh.cwnu-4D* region were extracted from the Chinese Spring reference genome (RefSeq v2.1) using the IntervalTools web server. Initially, 47 high-confidence genes were identified. After fine-mapping using KASP marker *K-cwnu-4D-502238348*, the region was narrowed down to 39 candidate genes. Of these, five genes were located within the 530 kb trichome-associated LD block previously defined in *Ae. tauschii* (Table S2). Upon examining the gene annotations, five genes within the interval were identified as potential candidates. These genes encode a cell-wall invertase (TraesCS4D03G0811800), an α/β-hydrolase superfamily protein (TraesCS4D03G0812100), a TIR-NBS-LRR disease-resistance protein (TraesCS4D03G0812400), an extracellular-matrix-binding protein (Ebh; TraesCS4D03G0812500), and the ClpB molecular chaperone (TraesCS4D03G0813100) (Table S2).

Notably, these genes were involved in cell wall modification, stress response, and disease resistance, which were consistent with the known roles of trichomes in plant defense and stress tolerance. Further analysis of the CM42 genome showed that only four high-confidence genes were predicted within the corresponding interval, and none corresponded to TraesCS4D03G0812500 (Table S3).

### 3.6. Structural Variation and Amplification Analysis Within QLsh.cwnu-4D

To investigate potential presence and absence variation in the fine-mapped interval, three reference assemblies were visualized, viz. *T. aestivum* Chinese Spring v2.1, *T. aestivum* CM42, and *Ae. tauschii* AL8/78 Aet5.0, in JBrowse. Between genes CM424D478000.1 and CM424D478100.1, an unannotated TIR-NBS-LRR disease-resistance gene was evident in CM42 and AL8/78 Aet5.0 genomes but missed in Chinese Spring v2.1 genome (Figure S5 and Table S3). However, despite multiple primer pairs, this locus failed to amplify from both genomic DNA and cDNA of ZKLM1, indicating its deletion or extensive divergence in this line (Figure S6).

The remaining four genes descending from CM424D478100.1 were then examined. In CM42, CM424D478300.1, orthologous to TraesCS4D03G0813100 in Chinese Spring, consists of four exons and three introns, whereas Chinese Spring RefSeq v2.1 annotates TraesCS4D03G0813100 as a single exon. To resolve this discrepancy, we loaded the high-quality CS-IAAS T2T assembly [28] into JBrowse and observed two identical paralogs, CSIAAS4DG0871000HC and CSIAAS4DG0871200HC, each with three exons and two introns (Figure S7). PCR amplification of this locus yielded the expected genomic fragment only in SM830, with no corresponding cDNA product, while ZKLM1 produced multiple non-specific products from both genomic DNA and cDNA (Figure S8). These data strongly suggest that the entire genomic region spanning the two TraesCS4D03G0813100 paralogs has been replaced or deleted in ZKLM1, positioning the causal gene either upstream or downstream of this structural variant.

To pinpoint the candidate region further, the 35 remaining high-confidence genes were screened for annotation and expression in *T. aestivum* Chinese Spring using WheatOmics 1.0. TraesCS4D03G0809500, encoding a 12-oxophytodienoate reductase-like protein and expressed in leaves, lay immediately upstream of the 530 kb trichome-associated LD block. Yet, only SM830 yielded the expected PCR products; ZKLM1 amplicons were abnormally large and, upon sequencing, proved non-target (Figure S9). This upstream deletion/replacement narrows the candidate interval to the region immediately downstream of the 530 kb LD block.

### 3.7. Identification and Sequence Analysis of TaSAIN1-4D

**Figure 5:**
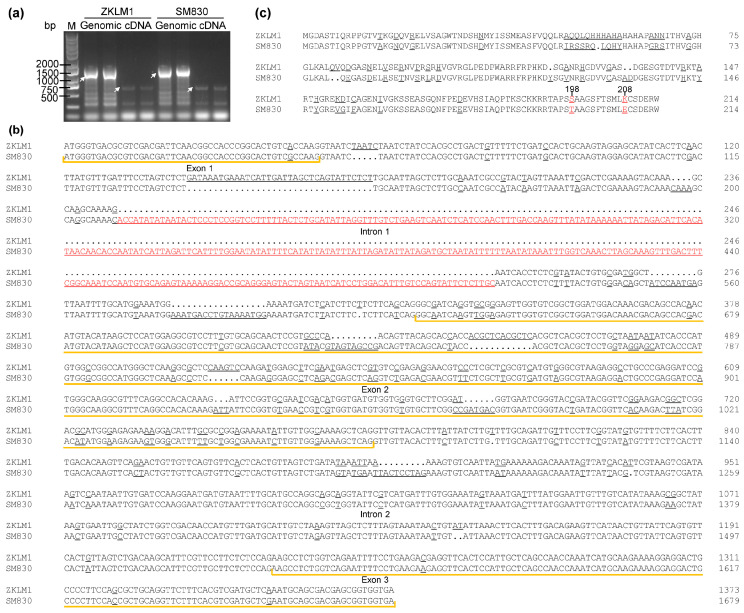
Isolation and characterization of the candidate gene *TaSAIN1-4D*. (**a**) PCR amplification of genomic DNA and CDS sequences of the candidate gene *TaSAIN1-4D*. (**b**) Nucleotide sequence alignments of *TaSAIN1-4D* between ZKLM1 and SM830. (**c**) Amino acid sequence alignments of *TaSAIN1-4D*. M indicates the molecular size marker. White arrowheads highlight the target PCR bands. Polymorphic sites are underlined, and critical polymorphic sites are highlighted in red font.

Analysis of 16 candidate genes downstream of the 530 kb LD block identified *TraesCS4D03G0814900*, a cold-regulated gene with high stem expression in *Chinese Spring*, as a potential target. PCR amplification of cDNA and genomic DNA from the parental lines ZKLM1 and SM830 produced a 0.6 kb band in both cDNA samples, and 1.4 kb and 1.6 kb bands in the genomic DNA of ZKLM1 and SM830, respectively (Figure 5a). Sequencing of the amplified fragments from SM830 confirmed the gene as a salt-inducible protein (SAIN1) gene in wheat, similar to the one in *Leymus chinensis* associated with salt stress tolerance (accession no. JX972110). In contrast, the amplified fragment from ZKLM1 genomic DNA exhibited only 68.6% sequence similarity with that of SM830, while the similarity for the CDS sequences was as high as 85.63%. A BLAST search in the NCBI database revealed less than 90% similarity to any wheat homologs, suggesting that this fragment likely originated from *Th. ponticum* rather than wheat. Additionally, no homologous genes were found in other grass species in the NCBI database.

Further analysis using the WheatOmics 1.0 platform revealed no homologous gene for this locus in *A. thaliana* and only 44.6% similarity to the homologous gene in rice. These findings suggest that the gene is likely unique to Triticeae species. Following the naming conventions used in the *Leymus* genus, it was designated *TaSAIN1-4D*. The homologous genes in ZKLM1 and SM830 were named *TaSAIN1-4Da* and *TaSAIN1-4Db*, respectively. Two splicing variants of *TaSAIN1-4Da* were identified and named *TaSAIN1-4Da.1* and *TaSAIN1-4Da.2* (Figure S10), and these sequences were deposited in the NCBI database (accession nos. PX116521-PX116523). The *TaSAIN1-4D* gene consists of three exons and two introns. Alignment of *TaSAIN1-4Da.1* and *TaSAIN1-4Db* revealed extensive genetic variation in the first intron and second exon. Specifically, 17 SNPs and 7 InDels were identified in the first intron, with the largest InDel measuring 311 bp and the smallest only 1 bp. In the second exon, 61 SNPs and 5 InDels were detected, resulting in 34 amino acid substitutions and 6 amino acid insertions or deletions. Additionally, one SNP in the first exon and three SNPs in the third exon led to one and three amino acid substitutions, respectively (Figure 5b,c).

The homologous gene sequence of *TaSAIN1-4D*, named *TaSAIN1-4Dc*, was extracted from the whole-genome sequencing assembly of cv. CM42 and aligned with *TaSAIN1-4Db*. The alignment identified 19 SNPs and 3 InDels, with an overall sequence similarity of 80.92%, while that for the CDS was as high as 99.38%. Four SNPs located in exons resulted in amino acid substitutions, while the three InDels, all situated in the first intron, were 3 bp, 11 bp, and 270 bp in length, respectively (Figure S11). These sequence variations, particularly the 270 bp InDel, suggest that the two gene variants may have distinct functions, which could explain the observed differences in LSH between CM42 and SM830.

### 3.8. Predicted Subcellular Localization of TaSAIN1-4D

**Figure 6:**
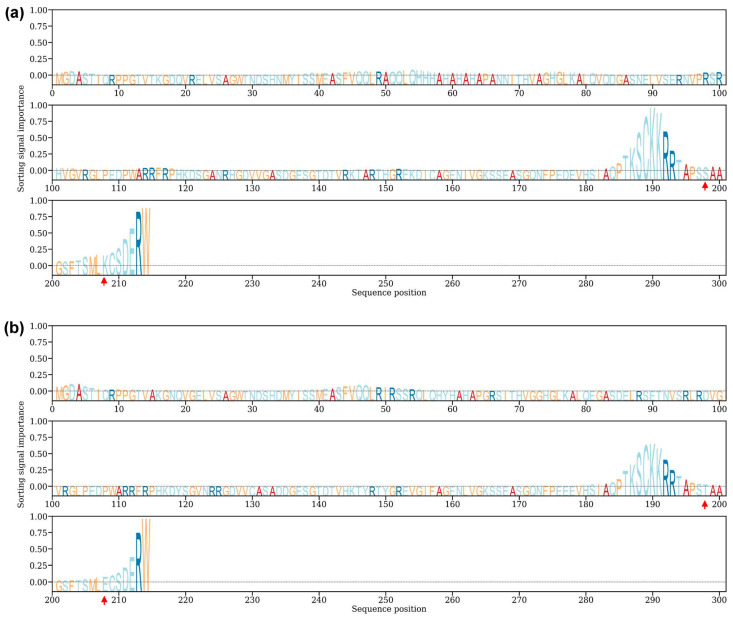
Sorting signal importance of the amino acid sequence of *TaSAIN1-4D* protein. (**a**) Sorting signal analysis of the *TaSAIN1-4Da*.1 protein. (**b**) Sorting signal analysis of the *TaSAIN1-4Db* protein. Red arrowheads highlight the acid substitution within the nuclear localization signal peptide fragment.

To predict the subcellular localization of the *TaSAIN1-4D* protein, SignalP v6.0 and DeepLoc v2.1 were used. SignalP analysis indicated the absence of a signal peptide, while DeepLoc predicted that *TaSAIN1-4Da*.1, *TaSAIN1-4Db*, and *TaSAIN1-4Dc* were most likely localized to the nucleus, with high probabilities of 0.9365, 0.8966, and 0.9478, respectively. All three proteins were also predicted to be associated with the soluble fraction, with probabilities of 0.8170, 0.8370, and 0.8630, respectively (Table 4). Sorting signal analysis identified nuclear localization signals in *TaSAIN1-4D*, supporting its predicted nuclear localization. Notably, the nuclear localization signal was stronger in ZKLM1 and CM42 than in SM830, potentially due to an S198T amino acid substitution in ZKLM1 and an I194T substitution in CM42, both relative to SM830. These substitutions occurred within the same nuclear localization signal peptide fragment (Figure 6 and Figure S12).

### 3.9. Comparative Analysis of TaSAIN1-4D and Known Trichome-Related Genes/QTLs

A comparative summary of *TaSAIN1-4D* with other known genes and QTLs involved in trichome development across cereals and model plants revealed substantial diversity in the molecular functions that regulated trichome formation (Table 5). *TaSAIN1-4D*, identified in wheat (4DL), encoded a salt-inducible nuclear protein unique to the Triticeae family, distinguishing it from other trichome-related genes. For instance, the *Hl1* locus in wheat and the *Hsh* gene in barley were responsible for leaf sheath hairiness, while *GL6*/*Mhl1* in rice and maize regulated trichome density and length. The *WOX (Hg)* transcription factor on wheat chromosome 1AS also contributed to trichome development. In addition, *GL1*, *GL3*, and *TTG1* in *Arabidopsis* formed the MYB–bHLH–WD40 (MBW) complex, a conserved mechanism for trichome initiation.

**Figure 7:**
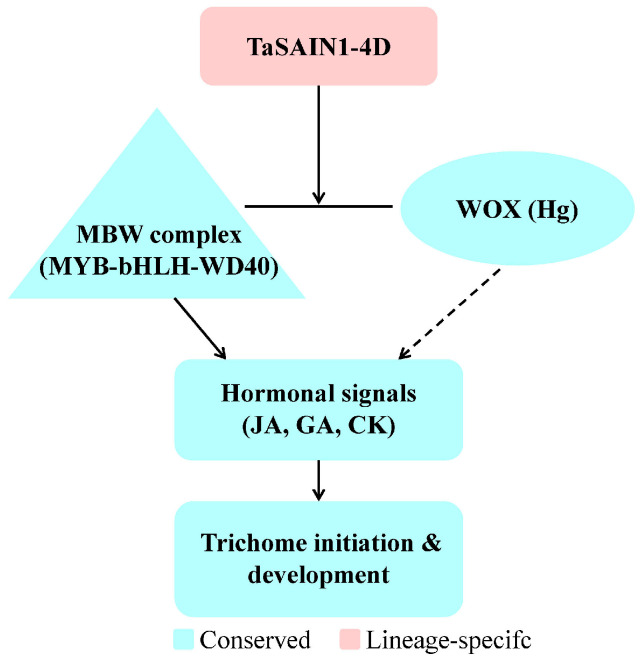
Proposed model of the role of *TaSAIN1-4D* in the wheat trichome regulatory network. *TaSAIN1-4D* functions as a central regulator that integrates signals from the MBW complex (MYB-bHLH-WD40), the wheat *WOX* (*Hg*) homolog, and hormonal pathways including JA, GA, and CK.

A model of the trichome regulatory network indicated that *TaSAIN1-4D* interacted with multiple regulatory components, including the MBW complex, the wheat *WOX (Hg)* homolog, and hormonal signals such as jasmonic acid (JA), gibberellins (GA), and cytokinins (CK), all of which collectively influenced trichome initiation and elongation (Figure 7). Within this framework, *TaSAIN1-4D* served as a central node that integrated stress cues, such as salt stress, with developmental pathways that govern epidermal differentiation. The proposed interactions positioned *TaSAIN1-4D* as a regulator of trichome formation with a potential role in stress adaptation.

## 4. Discussion

### 4.1. Genetic Basis of Leaf Sheath Hairiness in Wheat

This study successfully identified and fine-mapped a major QTL for LSH trait in wheat, *QLsh.cwnu-4D*, to a 1.67 Mb interval on chromosome 4DL. This region shares high collinearity with a previously described 530 kb LD block in *Ae. tauschii*, underscoring the evolutionary conservation of this trait within the Triticeae subfamily [8,15,34]. The complementary BSA-seq algorithms (Euclidean-distance, *G′*, and Δ(SNP-index)) used in this study provided high-resolution mapping power, comparable to approaches applied in QTL mapping in crops such as rice and maize [35,36]. The identification of this locus in both wheat and its wild relatives suggests that the genetic control of LSH has been conserved across species, making it a valuable model for understanding similar traits in related cereals.

### 4.2. Comparative Genomics

The *QLsh.cwnu-4D* interval was refined to 1.67 Mb with KASP marker *K-cwnu-4D-502238348*, overlapping the 530 kb LD block previously associated with marginal trichome number in *Ae. tauschii* [15]. This overlap suggests conservation of trichome-regulatory loci within the D genome lineage. The hairy parental line ZKLM1 traces part of its genetic background to *Th. ponticum*, raising the possibility that *QLsh.cwnu-4D* represents an introgressed haplotype conferring enhanced trichome development. This interpretation aligns with earlier successes in introgressing alleles such as *Hl1^th^* from *Th. ponticum* into elite wheat backgrounds [16].

Sequence alignment of *TaSAIN1-4D* revealed the genomic DNA and CDS sequence similarities between SM830 (*TaSAIN1-4Db*) and ZKLM1 (*TaSAIN1-4Da.1*) of 68.6% and 85.63%, respectively, while the similarities between SM830 (*TaSAIN1-4Db*) and CM42 (*TaSAIN1-4Dc*) for genomic DNA and CDS sequences were higher, at 80.92% and 99.38%, respectively. This divergence also explains why the three candidate genes, namely an unannotated TIR-NBS-LRR gene, TraesCS4D03G0813100, and TraesCS4D03G0809500, located within or upstream of the 530 kb trichome-associated LD block, could not be correctly amplified to the expected target bands (Figures S6, S8 and S9). These observations support the hypothesis that *QLsh.cwnu-4D* may have been introduced along with the blue grain gene (also located on 4DL) during the wheat–*Th. ponticum* translocation process.

Similar structural changes associated with agronomic traits like grain hardness and awn length have been documented previously [37,38,39]. In addition, *TaSAIN1-4Db* exhibited significant haplotypic variation, further refining the candidate region downstream of the 530 kb LD block. The presence of 311 bp and 270 bp insertions in the first intron of *TaSAIN1-4Db* compared to *TaSAIN1-4Da.1* and *TaSAIN1-4Dc*, respectively, suggests that these insertions may influence its gene function, potentially altering transcriptional regulation and downstream signaling pathways involved in trichome formation.

### 4.3. Relationship to Stress Tolerance

Among the genes located within the refined candidate region, TraesCS4D03G0814900 (*TaSAIN1-4D*) emerges as a potential key regulator of LSH. This salt-inducible protein, specific to the Triticeae lineage, shows minimal homology to *Arabidopsis* proteins and only partial similarity (44.6%) with rice counterparts, highlighting its role in a lineage-specific adaptation [40,41]. The 311 bp and 270 bp insertion identified in *TaSAIN1-4Db* may alter its function, possibly affecting both trichome development and stress responses.

Previous studies have shown that salt-inducible proteins can interact with transcription factors such as bHLH to influence epidermal differentiation under saline conditions [42]. This is consistent with the idea that *TaSAIN1-4D* may mediate an alternative regulatory pathway in grasses, distinct from the well-characterized MYB–bHLH–WD40 complex found in *Arabidopsis* [29,30,31,43].

### 4.4. Proposed Mechanism

Extensive research in *A. thaliana* has established a core regulatory network for trichome development. This involves the MYB–bHLH–WD40 transcriptional activator complex, comprising the R2R3 MYB factor GLABRA1 (GL1) [29], the bHLH proteins GLABRA3 (GL3) and ENHANCER OF GLABRA3 (EGL3) [30,43], and the WD40-repeat protein TRANSPARENT TESTA GLABRA1 (TTG1) [31]—which together induce GLABRA2 (GL2), a homeodomain–leucine zipper factor essential for trichome differentiation [44,45]. Negative regulators such as TRIPTYCHON (TRY) and CAPRICE (CPC) fine-tune trichome density and spacing [32,46,47,48]. This well-defined framework provides a valuable reference point for exploring both conserved and divergent aspects of trichome regulation in cereals.

These findings suggest that while homologs of MYB and bHLH proteins may contribute to trichome development in wheat [7,9], an alternative pathway mediated by the stress-responsive gene *TaSAIN1-4D* is also likely involved. The nuclear localization of *TaSAIN1-4D* implies a role as a transcriptional co-regulator, potentially modulating genes related to cell-wall remodeling and stress signaling that facilitate trichome outgrowth. Moreover, its potential interactions with MYB or bHLH factors could integrate developmental and environmental cues, thereby regulating trichome initiation under both normal and stress conditions. This dual role, linking trichome development with abiotic stress responses, parallels the function of other wheat transcription factors, such as DREB, which mediate both drought adaptation and trichome density [33,49].

Together, these insights support a proposed mechanism in which wheat trichome development is shaped not only by canonical MYB–bHLH–WD40 complexes but also by lineage-specific, stress-inducible regulators like *TaSAIN1-4D*. This highlights a potentially unique regulatory architecture in cereals, offering new avenues for functional validation and crop improvement.

### 4.5. Implications for Wheat Breeding

The co-dominant KASP marker *K-cwnu-4D-502238348*, which perfectly cosegregates with LSH in 92 F_2:3_ family lines, provides a powerful tool for marker-assisted selection (MAS) in wheat breeding programs. The inclusion of *TaSAIN1-4D* alleles in elite wheat germplasm could improve stress resilience, particularly in environments prone to drought and salinity, without negatively affecting yield potential. Previous studies have shown that introgressions of stress-responsive QTL, such as those from *Aegilops* or *Thinopyrum*, have successfully enhanced drought tolerance in durum wheat and other crops [50]. Future work will explore the potential for pyramiding *TaSAIN1-4D* with other trichome-modulating QTL to further optimize pubescence traits for specific environmental conditions.

### 4.6. Future Directions

To validate the function of *TaSAIN1-4D*, transgenic overexpression and CRISPR/Cas9-mediated knockouts in wheat backgrounds such as CM42 and ZKLM1 will be necessary. These approaches will provide direct evidence of the role of *TaSAIN1-4D* in trichome formation and stress tolerance. Additionally, transcriptomic and chromatin-immunoprecipitation (ChIP) analyses will help identify downstream target genes and potential interacting partners. Investigating natural allelic variation in *TaSAIN1-4D* across global wheat panels may reveal superior haplotypes with enhanced stress tolerance. Integrating high-resolution phenotyping platforms will also enable precise quantification of trichome density and its associated physiological effects under field conditions, further advancing our understanding of the role of leaf pubescence in stress adaptation.

## 5. Conclusions

This study successfully identified and fine-mapped a major QTL, *QLsh.cwnu-4D*, associated with LSH in wheat, providing valuable insights into the genetic basis of this adaptive trait. The refined *QLsh.cwnu-4D* region on 4DL overlaps with a trichome-associated LD block in *Ae. tauschii*, suggesting evolutionary conservation of trichome-regulatory genes within the Triticeae subfamily. The gene *TaSAIN1-4D*, identified within this region, is a salt-inducible protein gene potentially involved in both trichome formation and stress response. Structural variations within *TaSAIN1-4D*, including significant insertions, may influence gene function and contribute to trait differentiation. These findings offer a deeper understanding of the genetic mechanisms regulating leaf pubescence and provide a foundation for developing wheat varieties with improved stress resilience. The co-dominant KASP marker *K-cwnu-4D-502238348* offers a promising tool for marker-assisted selection in wheat breeding programs, enabling the introgression of stress-resilient traits into elite cultivars. Future research will focus on validating the functional role of *TaSAIN1-4D* through gene editing and exploring its potential in breeding strategies to optimize stress tolerance and pubescence traits for diverse environmental conditions.

## Figures and Tables

**Table 1:** Filtering and statistical analysis of the 120K-4HWA targeted-capture SNP sequencing data.

Samples	Raw Bases (bp)	Raw Reads (bp)	Clean Bases (bp)	Clean Reads (bp)	Clean Bases (%)	Clean Reads (%)	Q30 ^1^ (%)	GC (%)	Duplication ^2^ (%)
LSH_pool1+	7,354,933,800	49,032,892	7,125,150,346	48,695,506	96.88	99.31	96.9	52.5	4.1
LSH_pool1−	8,657,837,700	57,718,918	8,390,741,334	57,334,828	96.91	99.33	96.7	52.6	3.9
LSH_pool2+	7,762,636,200	51,750,908	7,606,138,898	51,401,226	97.98	99.32	96.1	52.9	4.0
LSH_pool2−	7,286,962,200	48,579,748	7,046,164,016	48,241,024	96.70	99.30	97.0	52.9	4.1
ZKLM1+	4,711,597,500	31,410,650	4,526,831,572	31,254,282	96.08	99.50	97.0	51.9	4.1
SM830−	4,432,805,400	29,552,036	4,250,976,330	29,393,964	95.90	99.47	96.9	52.3	3.5
SM2262−	4,359,853,500	29,065,690	4,211,924,814	28,922,050	96.61	99.51	96.7	51.1	3.9

^1^ Q30 (%) denotes the percentage of bases with a Phred score > 30; ^2^ Duplication (%) indicates the proportion of duplicated reads. LSH_pool1+ and LSH_pool1- are pools of F_2_ individuals from the SM830 × ZKLM1 cross with (+) or without (−) LSH, respectively; LSH_pool2+ and LSH_pool2− are the corresponding pools from the SM2262 × ZKLM1 cross.

**Table 2:** Alignment statistics for the targeted-capture sequencing data.

Samples	Mapping Rate ^1^ (%)	Average Depth ^2^ (×)	Coverage ^3^ ≥ 4× (%)	Coverage ≥ 10× (%)	Coverage ≥ 30× (%)	Capture Rate ^4^ (%)
LSH_pool1+	99.99	60.46	97.16	94.33	73.11	31.30
LSH_pool1−	99.99	69.97	97.23	94.59	77.69	31.15
LSH_pool2+	99.99	59.74	97.26	94.46	72.89	31.12
LSH_pool2−	99.99	59.91	97.18	94.11	72.53	30.85
ZKLM1+	99.99	37.62	94.65	87.59	48.52	28.53
SM830−	99.99	36.55	94.50	87.45	47.68	29.89
SM2262−	99.99	28.09	93.17	82.16	32.48	22.84

^1^ Mapping rate (%) refers to the proportion of reads aligned to the reference genome relative to the total number of reads; ^2^ Average depth (×) is the ratio of the total number of bases aligned to the core region to the total number of bases in the core region; ^3^ Coverage ≥ 4×, ≥10×, or ≥30× denotes the percentage of target regions covered by at least 4, 10, or 30 reads, respectively; ^4^ Capture rate (%) indicates the proportion of reads that fall within the capture area relative to the total number of reads in that region.

**Table 3:** Comparison of QTLs identified in this study with previously published QTLs.

Populations	QTLs/LD Block	Peak Intervals	Positions ^1^ (Mb)	References
C8 RILs	*QLsh.saas-4D*	Xbarc48—D_GB5Y7FA01AGCKH_224	504.32–511.67	[8]
CC RILs	*QLsh.saas-4D*	Xbarc48–Xbarc1183	504.32–517.02	[8]
*Ae. tauschii* accessions	*LD block-trichomes-4DL*	Between 530 kb LD block on 4DL	502.84–503.26	[15]
SM830/ZKLM1 F_2_	*QLsh.cwnu-4D*	n.501803422–n.503905439	501.80–503.91	This study
SM2262/ZKLM1 F_2_	*QLsh.cwnu-4D*	n.501803422–n.503905382	501.80–503.91	This study

^1^ Positions, physical positions are based on the Chinese Spring RefSeq v2.1 genome.

**Table 4:** Predicted subcellular localization and membrane-association type of *TaSAIN1-4D* protein.

Cultivars	Probabilities of Localization (Top 4)				Probabilities of Membrane Association Type			
	Cytoplasm	Nucleus	Mitochondrion	Peroxisome	Peripheral	Transmembrane	Lipid anchor	Soluble
ZKLM1	0.2662	0.9365	0.1483	0.1795	0.3550	0.1420	0.0870	0.8170
SM830	0.2820	0.8966	0.1186	0.3141	0.3080	0.1090	0.0720	0.8370
CM42	0.2122	0.9478	0.1061	0.4360	0.2540	0.0950	0.0450	0.8630

**Table 5:** Comparative summary of *TaSAIN1-4D* and known trichome-related genes/QTLs across cereals and model plants.

Gene/QTL	Species/Locus	Molecular Type and Function	References
*TaSAIN1-4D*	Wheat (4DL)	Salt-inducible nuclear protein; unique to Triticeae	This study
*Hl1*/*Hl1^th^*	Wheat (4BL)/*Th. ponticum* introgression	Dominant locus for leaf-blade/sheath pubescence	[16]
*QLsh.saas-4D*	Wheat (4DL)	QTL for hairy leaf sheath (LSH)	[8]
*Hsh*	Barley (4HL)	Gene controlling leaf sheath hairiness (Hsh)	[9]
*Hp1*	Rye (5RL)	Hairy peduncle gene (Hp)	[9]
*GL6*/*Mhl1*	Rice (Chr. 6)/Maize (Chr. 9)	Homologous loci regulating trichome density/length	[9]
*Hg* (*WOX3-like*)	Wheat (1AS)	WOX transcription factor	[9]
*GL1* (*R2R3-MYB*)	*Arabidopsis*	Activator of trichome initiation (MBW complex)	[29]
*GL3*/*EGL3* (*bHLH*)	*Arabidopsis*	Partners with GL1 and TTG1; activates GL2	[30]
*TTG1* (*WD40*)	*Arabidopsis*	WD40 scaffold protein	[31]
*TRY*/*CPC* (*R3-MYBs*)	*Arabidopsis*	Mobile negative regulators of trichome density	[32]
*DREB* family	Wheat (Various loci)	AP2/ERF transcription factors	[33]

## Data Availability

The original contributions presented in this study are included in the article. Further inquiries can be directed toward the corresponding authors.

## Supplementary Materials

The following supporting information can be downloaded at: https://www.mdpi.com/article/10.3390/genes16091117/s1, Figure S1: Phenotypic analysis of the leaf auricle. (**a**) SM830; (**b**) SM2262; (**c**) ZKLM1; (**d**) F_1_ hybrid of the SM830/ZKLM1 cross; (**e**) and (**f**) leaf auricle phenotypes in the SM830/ZKLM1 F_2_ population; (**g**) F_1_ hybrid of the SM2262/ZKLM1 cross; (**h**) and (**i**) leaf auricle phenotypes in the SM2262/ZKLM1 F_2_ population. The red arrow indicates auricle hairiness; Figure S2: Phenotypic analysis of the leaf blade. (**a**) SM830; (**b**) SM2262; (**c**) ZKLM1; (**d**) F_1_ hybrid of the SM830/ZKLM1 cross; (**e**) and (**f**) leaf blade phenotypes in the F_2_ population of the SM830/ZKLM1 cross; (**g**) F_1_ hybrid of the SM2262/ZKLM1 cross; (**h**) and (**i**) leaf blade phenotypes in the F_2_ population of the SM2262/ZKLM1 cross; Figure S3: QTL mapping of the LSH trait in SM2262/ZKLM1 bulks using Euclidean distance (ED)- and *G′*-based metrics. (**a**) Manhattan plot of unfitted ED^4^ and locally fitted ED^4^ values across the 21 wheat chromosomes, highlighting peaks corresponding to trait-associated loci. (**b**) Genome-wide *G′* profile showing smoothed allele frequency divergence between bulks. (**c**) A magnified view of the *G′* profile for the distal region of chromosome 4D. *G′* statistics were computed from SNP-wise G values using a sliding window smoothing method. Significance thresholds (*G′* ≥ 4.263; *n* = 84) were assessed using empirical permutation testing and indicated using green dotted line; Figure S4: Δ(SNP-index)–based QTL analysis of the LSH trait in mixed SM2262/ZKLM1 bulks. (**a**) Genome-wide Δ(SNP-index) profile across all 21 wheat chromosomes. (**b**) An enlarged view of the distal region of chromosome 4D highlights a major QTL peak. The blue line denotes Δ(SNP-index); green and yellow lines represent the 95% and 99% confidence intervals (CI), respectively. CI (*n* = 84) were determined via simulation-based null distribution modeling. Significant Δ(SNP-index) peaks exceeding the 95% or 99% CI indicate potential QTLs. The red dotted line marks the genomic interval most strongly associated with LSH; Figure S5: JBrowse visualization of the genomic interval between TraesCS4D03G0811800 and TraesCS4D03G0812100 in three reference assemblies. (**a**) *T. aestivum* ‘Chinese Spring’ v2.1, (**b**) *T. aestivum* ‘Chuanmai 42’, and (**c**) *Ae. tauschii* cv. AL8/78 Aet5.0; Figure S6: PCR amplification of the genomic and CDS sequences of the unannotated gene encoding a disease-resistant protein. M, molecular size marker; Figure S7: JBrowse visualization of the allele structure of TraesCS4D03G0813100 in three reference assemblies. (**a**) *T. aestivum* ‘Chinese Spring’ v2.1, (**b**) *T. aestivum* ‘Chuanmai 42’, and (**c**) *T. aestivum* ‘Chinese Spring’ CS-IAAS T2T; Figure S8: PCR amplification of the genomic and CDS sequences of TraesCS4D03G0813100. M, molecular size marker; Figure S9: PCR amplification of the genomic and CDS sequences of TraesCS4D03G0809500 encoding a 12-oxophytodienoate reductase-like protein. M, molecular size marker. The white arrowheads indicate the target PCR bands; Figure S10: Alignment of two splicing variants of *TaSAIN1-4Da*; Figure S11: Alignments of *TaSAIN1-4D* between CM42 and SM830. (**a**) Nucleotide sequence alignments of *TaSAIN1-4D*. (**b**) Amino acid sequence alignments of *TaSAIN1-4D*. Ellipses represent the omission of the consensus sequence; Figure S12: Sorting signal importance of *TaSAIN1-4D* protein. (**a**) Sorting signal analysis of the *TaSAIN1-4Dc* protein. (**b**) Sorting signal analysis of the *TaSAIN1-4Db* protein. Red arrowheads highlight the acid substitution within the nuclear localization signal peptide fragment; Table S1: Primers used in this study; Table S2: High-confidence candidate genes within the *QLsh.cwnu-4D* interval (RefSeq v2.1); Table S3: High-confidence candidate genes within the 530-kb LD block of the Chuanmai 42 genome.

## Abbreviations

The following abbreviations are used in this manuscript:

BSA-seq	Bulk segregant analysis sequencing
QTL	Quantitative trait locus
GWAS	Genome wide association study
ED	Euclidean distance
KASP	Kompetitive Allele Specific PCR
MAS	Marker-assisted selection
SNP	Single nucleotide polymorphism
InDel	Insertion and Deletion
CDS	coding sequence
cDNA	complementary DNA
LD block	linkage disequilibrium block
RILs	Recombinant inbred lines
