# Supplementary material for: Fine Mapping of a Major Locus for Leaf Sheath Hairiness in Wheat Identifies TaSAIN1-4D as a Candidate Gene
Source: Genes (Basel). 2025 Sep 20;16(9):1117. doi: 10.3390/genes16091117 (PMC12469731; doi:10.3390/genes16091117)
Supplement: Supplementary file 1 [file genes-16-01117-s001.zip › Table S.pdf]

Table S1. Primers used in this study.

Primers	Sequences (From 5´ to 3´)	Tm (°C)	Use
K-cwnu-4D-502238348-G	GAAGGTGACCAAGTTCATGCTaggttgttgtagtcggctcG	61-55 (-0.6 °C/cycle)	KASP marker
K-cwnu-4D-502238348-T	GAAGGTCGGAGTCAACGGATTaggttgttgtagtcggctcT		
K-cwnu-4D-502238348-Com	ctgtcaccacagttcggact		
TaSAIN1-4D-F	TTGGTCCGATGGGTGACGCGTCGAC	64	TaSAIN1 amplification
TaSAIN1-4D-R	TCACCACCGCTCGTCGCTGCATTCGA		

Table S2. High-confidence candidate genes within the *QLsh.cwnu-4D* interval (RefSeq v2.1).

Chr.	Start (Mb)	End (Mb)	Gene03G	Strand	Function description	Domain
4D	502.146539	502.149138	TraesCS4D03G0807300	+	L-allo-threonine aldolase	PF01212: Beta-eliminating lyase
4D	502.150007	502.154589	TraesCS4D03G0807400	-	G patch domain protein	null
4D	502.16381	502.164346	TraesCS4D03G0807500	-	Dehydration-responsive element binding factor	PF00847: AP2 domain
4D	502.166752	502.169332	TraesCS4D03G0807600	+	Pentatricopeptide repeat-containing protein	PF13041: PPR repeat family; PF01535: PPR repeat; PF14432: DYW family of nucleic acid deaminases
4D	502.169204	502.173617	TraesCS4D03G0807700	-	Kinase, putative	PF03881: Fructosamine kinase
4D	502.19843	502.199167	TraesCS4D03G0808000	-	Xyloglucan endotransglucosylase/hydrolase	PF00722: Glycosyl hydrolases family 16
4D	502.199211	502.201374	TraesCS4D03G0808100	-	Riboflavin biosynthesis protein ribD	PF00383: Cytidine and deoxycytidylate deaminase zinc-binding region
4D	502.205121	502.207257	TraesCS4D03G0808200	+	Peroxisomal membrane protein 11 homolog	PF05648: Peroxisomal biogenesis factor 11 (PEX11)
4D	502.241744	502.243438	TraesCS4D03G0808500	-	U-box domain-containing protein 4	PF00514: Armadillo/beta-catenin-like repeat
4D	502.511021	502.513897	TraesCS4D03G0808700	+	Embryogenesis transmembrane protein-like	PF13962: Domain of unknown function
4D	502.52707	502.534359	TraesCS4D03G0808800	+	receptor kinase 1	PF02362: B3 DNA binding domain; PF01419: Jacalin-like lectin domain; PF00069: Protein kinase domain; PF00635: MSP (Major sperm protein) domain
4D	502.538856	502.541333	TraesCS4D03G0809000	+	4-hydroxy-tetrahydrodipicolinate reductase	PF01113: Dihydrodipicolinate reductase, N-terminus; PF05173: Dihydrodipicolinate reductase, C-terminus
4D	502.541649	502.544327	TraesCS4D03G0809100	-	26S proteasome non-ATPase regulatory subunit-like protein	PF01399: PCI domain
4D	502.545938	502.550578	TraesCS4D03G0809200	-	F-box family protein, putative, expressed	null
4D	502.555794	502.55665	TraesCS4D03G0809300	-	Cysteine-rich receptor-kinase-like protein	PF01657: Salt stress response/antifungal
4D	502.609714	502.612782	TraesCS4D03G0809400	+	Cell number regulator 6	PF04749: PLAC8 family
4D	502.618323	502.620215	TraesCS4D03G0809500	+	12-oxophytodienoate reductase-like protein	PF00724: NADH:flavin oxidoreductase / NADH oxidase family
4D	502.662992	502.666334	TraesCS4D03G0809600	+	Phytochelatin synthase	PF05023: Phytochelatin synthase; PF09328: Domain of unknown function (DUF1984)
4D	502.666519	502.668062	TraesCS4D03G0809700	-	Aquaporin-like protein	PF00230: Major intrinsic protein
4D	502.676228	502.678105	TraesCS4D03G0809900	-	Rhodanese-related sulfurtransferase	PF00581: Rhodanese-like domain
4D	502.683191	502.683929	TraesCS4D03G0810000	+	Defensin	PF00304: Gamma-thionin family
4D	502.688400	502.690978	TraesCS4D03G0810100	-	F-box protein PP2-A13	PF14299: Phloem protein 2
4D	502.699781	502.70014	TraesCS4D03G0810200	-	Asparagine synthetase	null
4D	502.700312	502.705459	TraesCS4D03G0810300	+	COP9 signalosome complex subunit 1	PF10602: 26S proteasome subunit RPN7; PF01399: PCI domain

Chr.	Start (Mb)	End (Mb)	Gene03G	Strand	Function description	Domain
4D	502.705908	502.708423	TraesCS4D03G0810400	-	Rhomboid-like protein	PF01694: Rhomboid family
4D	502.748216	502.750050	TraesCS4D03G0811000	+	Axonemal dynein light intermediate polypeptide 1	null
4D	502.836873	502.840769	TraesCS4D03G0811800	+	Cell wall invertase	PF00251: Glycosyl hydrolases family 32 N-terminal; PF08244: Glycosyl hydrolases family 32 C terminal
4D	502.875282	502.879444	TraesCS4D03G0812100	-	Alpha/beta-Hydrolases superfamily protein	PF00561: alpha/beta hydrolase fold
4D	502.888111	502.889392	TraesCS4D03G0812400	-	Disease resistance protein (TIR-NBS-LRR class)	PF00931: NB-ARC domain
4D	502.9946	502.995616	TraesCS4D03G0812500	+	Extracellular matrix-binding protein ebh	null
4D	503.268474	503.269244	TraesCS4D03G0813100	+	Chaperone protein ClpB	null
4D	503.276253	503.278891	TraesCS4D03G0813200	-	KHG/KDPG aldolase	PF01081: KDPG and KHG aldolase
4D	503.29358	503.294449	TraesCS4D03G0813300	-	O-methyltransferase	PF00891: O-methyltransferase
4D	503.29953	503.301215	TraesCS4D03G0813500	+	Chalcone synthase	PF00195: Chalcone and stilbene synthases, N-terminal domain; PF02797: Chalcone and stilbene synthases, C-terminal domain
4D	503.382312	503.387115	TraesCS4D03G0813700	-	Membrane protein insertase YidC	PF02096: 60Kd inner membrane protein
4D	503.477957	503.481561	TraesCS4D03G0814000	-	Palmitoyl protein thioesterase containing protein, expressed	PF02089: Palmitoyl protein thioesterase
4D	503.530973	503.533091	TraesCS4D03G0814200	+	Zinc finger (C3HC4-type RING finger) protein family-like	PF00092: von Willebrand factor type A domain; PF14624: VWA / Hh protein intein-like
4D	503.571944	503.573704	TraesCS4D03G0814300	+	Short-chain dehydrogenase/reductase family protein	PF00106: short chain dehydrogenase
4D	503.625956	503.627615	TraesCS4D03G0814500	-	Short-chain dehydrogenase/reductase family protein	PF00106: short chain dehydrogenase
4D	503.683388	503.68591	TraesCS4D03G0814800	+	Cytochrome P450, putative	PF00067: Cytochrome P450
4D	503.717037	503.719188	TraesCS4D03G0814900	-	Cold regulated protein 27	null
4D	503.727011	503.727969	TraesCS4D03G0815100	+	FAD-binding Berberine family protein, putative	PF08031: Berberine and berberine like
4D	503.735525	503.741658	TraesCS4D03G0815200	+	Respiratory burst oxidase, putative	PF08414: Respiratory burst NADPH oxidase; PF01794: Ferric reductase like transmembrane component; PF08022: FAD-binding domain; PF08030: Ferric reductase NAD binding domain
4D	503.74201	503.744091	TraesCS4D03G0815300	-	F-box family protein	PF12937: F-box-like
4D	503.787731	503.789907	TraesCS4D03G0815500	+	CASP-like protein	PF04535: Domain of unknown function (DUF588)
4D	503.791419	503.794602	TraesCS4D03G0815600	-	Receptor-kinase, putative	PF08263: Leucine rich repeat N-terminal domain; PF13855: Leucine rich repeat; PF00069: Protein kinase domain

Chr.	Start (Mb)	End (Mb)	Gene03G	Strand	Function description	Domain
4D	503.861128	503.866586	TraesCS4D03G0816200	+	Ankyrin repeat protein family-like protein	PF12796: Ankyrin repeats (3 copies); PF13962: Domain of unknown function

Table S3. High-confidence candidate genes within the 530-kb LD block of the Chuanmai 42 genome.

Chr.	Start (Mb)	End (Mb)	Gene	Strand	Function description	Domain
4D	514.285680	514.289226	CM424D478000.1	+	Cell wall invertase	PF00251: Glycosyl hydrolases family 32 N-terminal; PF08244: Glycosyl hydrolases family 32 C-terminal
4D	514.373090	514.378215	Unannotated	+	Disease resistance protein (TIR-NBS-LRR class)	PF00931: NB-ARC domain; PF23598: LRR_14
4D	514.424193	514.428074	CM424D478100.1	-	Alpha/beta-Hydrolases superfamily protein	PF00561: alpha/beta hydrolase fold
4D	514.431709	514.439589	CM424D478200.1	-	Disease resistance protein (TIR-NBS-LRR class)	PF00931: NB-ARC domain
4D	514.485133	514.486149	Unannotated	+	Extracellular matrix-binding protein ebh	null
4D	514.782537	514.787734	CM424D478300.1	+	Chaperone protein ClpB	Null
